# Multicellular Architecture of Malignant Breast Epithelia Influences Mechanics

**DOI:** 10.1371/journal.pone.0101955

**Published:** 2014-08-11

**Authors:** Gautham Venugopalan, David B. Camarillo, Kevin D. Webster, Clay D. Reber, James A. Sethian, Valerie M. Weaver, Daniel A. Fletcher, Hana El-Samad, Chris H. Rycroft

**Affiliations:** 1 Department of Bioengineering and Biophysics Program, University of California, Berkeley, California, United States of America; 2 Department of Bioengineering, Stanford University, Stanford, California, United States of America; 3 Department of Biochemistry & Biophysics, University of California San Francisco, San Francisco, California, United States of America; 4 Department of Mathematics, University of California, Berkeley, California, United States of America; 5 Department of Mathematics, Lawrence Berkeley Laboratory, Berkeley, California, United States of America; 6 Departments of Surgery, Anatomy, Bioengineering, and Therapeutic Sciences, University of California San Francisco, San Francisco, California, United States of America; 7 Center for Bioengineering and Tissue Regeneration, University of California San Francisco, San Francisco, California, United States of America; 8 Physical Biosciences Division, Lawrence Berkeley Laboratory, Berkeley, California, United States of America; 9 School of Engineering and Applied Sciences, Harvard University, Cambridge, Massachusetts, United States of America; University of California, Merced, United States of America

## Abstract

Cell–matrix and cell–cell mechanosensing are important in many cellular processes, particularly for epithelial cells. A crucial question, which remains unexplored, is how the mechanical microenvironment is altered as a result of changes to multicellular tissue structure during cancer progression. In this study, we investigated the influence of the multicellular tissue architecture on mechanical properties of the epithelial component of the mammary acinus. Using creep compression tests on multicellular breast epithelial structures, we found that pre-malignant acini with no lumen (MCF10AT) were significantly stiffer than normal hollow acini (MCF10A) by 60%. This difference depended on structural changes in the pre-malignant acini, as neither single cells nor normal multicellular acini tested before lumen formation exhibited these differences. To understand these differences, we simulated the deformation of the acini with different multicellular architectures and calculated their mechanical properties; our results suggest that lumen filling alone can explain the experimentally observed stiffness increase. We also simulated a single contracting cell in different multicellular architectures and found that lumen filling led to a 20% increase in the “perceived stiffness” of a single contracting cell independent of any changes to matrix mechanics. Our results suggest that lumen filling in carcinogenesis alters the mechanical microenvironment in multicellular epithelial structures, a phenotype that may cause downstream disruptions to mechanosensing.

## Introduction

**Figure 1:**
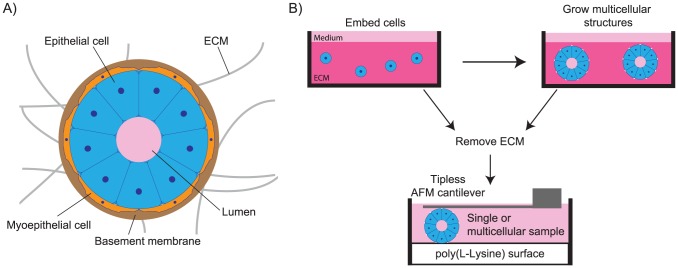
Background and experimental design. (A) A mammary epithelial cell grows in a dynamic environment surrounded by extracellular matrix, fluids, and other cells. (B) Mammary epithelial cells grown in laminin-rich extracellular matrix can be extracted and mechanically probed at single and multicellular states using identical trypsin-free extraction methods.

Epithelial cells reside in an environment where they are surrounded by other cells, extracellular matrix (ECM), and fluids. For example, in the mammary acinus, epithelial cells form a spherical shell with a hollow fluid-filled lumen, and are surrounded by other cells and ECM (Fig. 1A). Disruption of the orderly arrangement of epithelial structures is associated with pathologies such as ductal carcinoma *in situ* in the breast, where the hollow lumen of these acinar structures fills with cells [1]. Lumen filling can occur in response to genetic mutations [2] or increased ECM stiffness [3], and the resultant pre-malignant growths can be a precursor to invasive carcinoma [4]. *In vivo* breast carcinomas are stiffer than the surrounding tissue [5], and the mechanical properties of the ECM have been shown to influence cancer progression [3], [6]–[8]. However, whether the mechanical properties of the epithelial component of the acinus change during cancer progression remains unknown.

Mechanical differences between individual non-malignant and malignant mammary epithelial cells are well appreciated, for example with respect to cells grown on glass and polystyrene surfaces [9]. Cells grown on glass and plastic substrates have very different mechanical properties than those grown on softer substrates [10], making it difficult to extrapolate these results to an acinus. In a multicellular configuration, cells are also interconnected via cell–cell contacts. These cell–cell contacts transmit nanonewton-scale forces [11], enabling mechanosensing [12] and guiding proper development [13], [14] and function [15] of the tissue. However, the relative contributions of the individual cells, cell–cell junctions, and lumen formation to the mechanical microenvironment of breast epithelial structures remain unknown.

The mechanics of multicellular tissue structures have been studied using a variety of techniques. For example, *Xenopus laevis* embryonic tissue [16] and murine sarcoma model tissues [17] under micropipette aspiration behave in a linear elastic fashion. The stiffness of mouse mammary organoids has been characterized using atomic force microscopy (AFM) [18]. However, an investigation of the contributors to multicellular mechanical properties in phenotypically normal (hollow lumen) and pre-malignant (filled lumen) acini has not been performed. Furthermore, it is not clear what, if any, influence the multicellular mechanical properties have on cancer progression.

Healthy and pre-malignant acini could have different mechanical properties as a result of single cell changes, cell–cell connection strength, and multicellular architecture. To investigate the relative contributions of these factors, we carried out *in situ* experiments using MCF10A (hollow lumen) and MCF10AT (filled lumen) mammary epithelial cells. We cultured MCF10A and MCF10AT cells in laminin-rich ECM gels, extracted acini, and performed creep compression tests using an AFM (Fig. 1B), which allowed us to quantify both their elastic and viscoelastic properties. Throughout the paper, we define “stiffness” to be associated with the elastic part of our measurements, characterizing the long-duration mechanical response, in the absence of any short-term viscoelastic effects. Our data indicate that lumen formation was associated with a decrease in stiffness, as MCF10AT acini were stiffer than MCF10A acini. These differences could not be explained by mechanical properties of single cells or multicellular structures tested before lumen formation. To study how changes in multicellular architecture influence bulk multicellular elasticity, we developed a three-dimensional mechanical simulation of an acinus and calibrated it using our experimental data. Our simulation predicts that the absence of the lumen in the MCF10AT acini could lead to increased stiffness consistent with the experimental results. Further simulations of single cell contraction within a hollow or filled acinar structure predict approximately a 20% increase in the perceived stiffness by that cell when the lumen is filled. This suggests an architectural reinforcement of the stiffening, possibly amplifying the tumorigenic mechanical signaling.

## Results

### Healthy and pre-malignant acini have different mechanical properties

**Figure 2:**
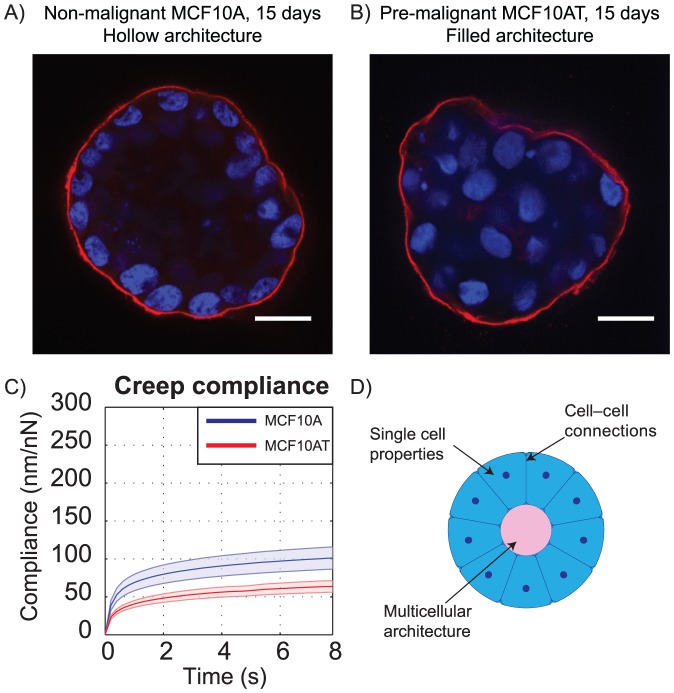
MCF10A and MCF10AT acini have different architectural and mechanical properties. Confocal immunofluorescence images of (A) non-malignant MCF10A (hollow lumen) and (B) pre-malignant MCF10AT (filled lumen) acini, taken after 15 days of growth. Scale bars 25 µm. (C) Creep compliance (mean ±95% CI) of hollow and filled breast epithelial acini. (*N* = 32 and 31 acini for A and T respectively) (D) Differences in mechanical response could be due to (1) different properties of single cells (2) changes in connectivity or (3) changes in multicellular architecture.

To measure the mechanical properties of healthy and pre-malignant acini, we used the MCF10A and MCF10AT model system. MCF10A cells are a human-derived breast epithelial cell line [19]. When embedded in laminin-rich ECM, MCF10A single cells grow into large structures with hollow lumina after a period of 2–3 weeks (Fig. 2A [20]). In contrast, c-Ha-*ras* transformed MCF10AT cells [21], [22] do not form lumina (Fig. 2B [2]). The MCF10A cell line has been previously used to demonstrate that breast epithelial cells sense ECM stiffness during acinar morphogenesis [3] and growth factor signaling [23].

Because mechanosensitive breast epithelial cells form filled lumen acini in response to both genetic mutations [2] and increased matrix stiffness [3], we hypothesized that healthy and pre-malignant acini could have different mechanical properties, which might provide a mechanical reinforcement of pre-malignancy. Given recent evidence that cell–cell junctions are mechanosensitive [12], the mechanics of the whole multicellular structure could play an important role in tumor formation. We developed a protocol that allowed us to extract cells from a laminin-rich ECM without protease digestion, therefore keeping intact structurally important proteins such as integrins and cadherins (Sec., Fig. 1B). Using an atomic force microscope (AFM), we applied step loads over the range of 10–50 nN to such isolated acini, and used force-feedback control to maintain a given load while recording deformation (Figure S1A–B). Both MCF10A and MCF10AT acini exhibited large initial displacements followed by viscous deformation (Fig. 2C). However, their responses were markedly different from each other: given the same environmental conditions and time to grow, pre-malignant MCF10AT acini were 1.6 times less compliant than phenotypically normal MCF10A acini (two-sided *t*-test, *p* = 5.5×10^−5^).

### Structural differences explain mechanical differences between healthy and pre-malignant acini

Three primary factors could account for the difference in compliance between MCF10A and MCF10AT acini (Fig. 2D): (1) single cell mechanics, (2) cell–cell connection strength, or (3) changes in multicellular architecture.

**Figure 3:**
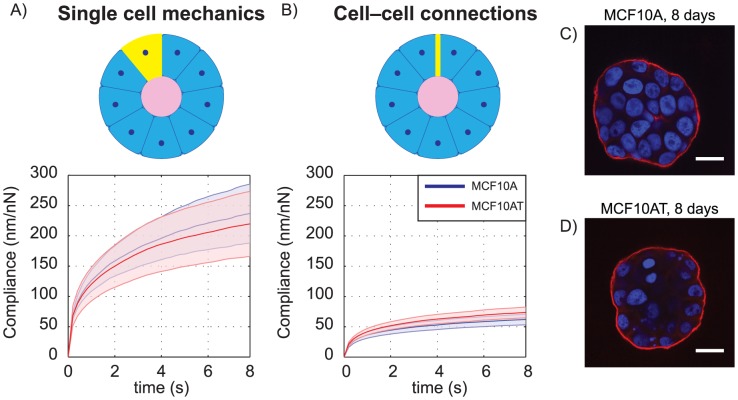
Single cell mechanics and cell–cell connections do not explain the mechanical differences. Creep compliance (mean ±95% CI) of MCF10A and MCF10AT cells at (A) single cell state (*N* = 14 and *N* = 15 cells for A and T respectively) and (B) 6–8 day state before lumen formation (*N* = 34 and *N* = 33 colonies for A and T respectively). Confocal immunofluorescence images of 8 day colonies of (C) MCF10A and (D) MCF10AT; 6–8 day time points were selected for testing because this was before lumina formed; scale bars 25 µm.

To test the potential contribution of mechanical changes at the single cell level, we embedded MCF10A and MCF10AT cells in laminin-rich ECM as before, but extracted them after twelve hours and subjected single cells to creep compression tests. MCF10AT single cells were not noticeably stiffer than MCF10A single cells (one-sided *t*-test, *p* = 0.329), suggesting that the increased stiffness observed for pre-malignant acini does not result from stiffer cells (Fig. 3A). To determine the influence of cell–cell connectivity strength, we extracted MCF10A and MCF10AT structures after 6–8 days of growth. As suggested by previous literature [20], 6–8 day-old MCF10A structures did not yet have lumina (*i.e.* acini were filled, Figure 3C,D). At this time point, healthy and pre-malignant structures had the same filled multicellular architecture, and did not exhibit distinguishable differences in creep compliance (Fig. 3B, *p* = 0.963). Since changes in cell–cell connectivity would be present at the 6–8 day time point, these data suggest that neither single cell mechanics nor cell–cell connectivity can account for the decreased compliance observed in pre-malignant structures. Notably, both of the 6–8 day filled structures (Fig. 3B) exhibited similar creep responses to mature MCF10AT acini (Fig. 2C). Assuming that cell–cell connectivity remains similar in the 6–8 day acini and the mature acini, the data suggests that the decreased stiffness of the acini coincides with hollow lumen formation.

### Predicted mechanical property changes due to structural differences are consistent with measurements

As the creep responses of MCF10A and MCF10AT were only different upon lumen formation, we developed a computational model to investigate how differences in multicellular architecture could affect the mechanical properties of the structure. Several high-accuracy models of single-cell mechanics are available in the literature, often employing a biphasic approach, in which the cell cytoplasm is considered to have both a solid phase and a fluid phase that interact [24]–[26]; similar approaches have also been used to model collagen networks [27], [28]. However, because our measurements are on the multicellular scale and probed small strains, we adopted a simpler modeling approach whereby the acinus is considered to be an incompressible linear viscoelastic solid immersed in an incompressible fluid. The boundary of the acinus is described using the level set method [29] (see Simulation Development).

**Figure 4:**
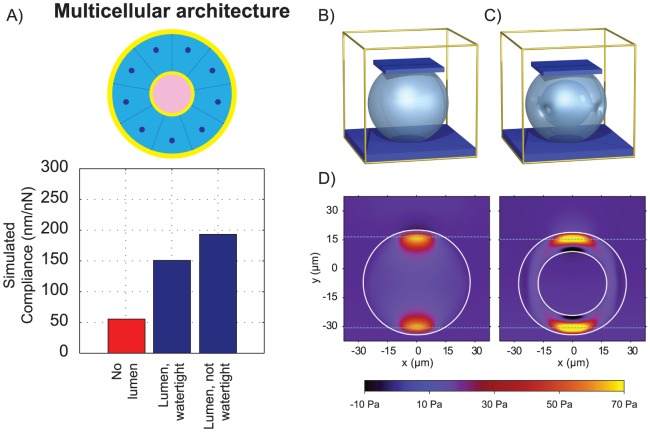
Mechanical differences are explained by the difference in multicellular architecture. (A) Simulation of hollow and filled structures predicts decreased compliance (increased stiffness) of the structure associated with multicellular architecture. (B,C) Visualization of the 3D plate compression simulation environment using in this study to model the MCF10AT and MCF10A acini geometries. The yellow cube represents the simulation domain which is filled with incompressible fluid. The light blue object represents the deforming elastic–viscoelastic acinus, which is compressed between the dark blue plates. (D) Cross-section through 3D simulation of plate for hollow and filled structures. For numerical convenience, the influences of the plate and bottom surface are smoothed out across several layers of grid points, so they appear to overlap with the top and bottom of the acini. Regions of higher pressure are visible at the locations where the plate and bottom surface make contact. In the spherical shell simulation, a region of negative pressure is also visible, as the interior part of the shell is stretched during the deformation.

To extract the simulation parameters from the mechanical displacement data, we formulated a standard linear solid (SLS) model, which has three parameters (Fig. S2). The simulation parameters were then uniquely fit using the MCF10AT data, and using a representative acinus diameter of 55 µm (see Simulation Development). To investigate the effects of multicellular structure alone, we simulated a filled sphere to approximate the MCF10AT geometry (Fig. 4B), and a hollow spherical shell to approximate the MCF10A geometry (Fig. 4C), using identical material properties. A representative shell thickness of 10 µm, based on a typical cell diameter and consistent with the literature [20] was used. The simulation allows us to model the deforming shape and stress distributions within the two acinus geometries (Fig. 4D). Our model predicted approximately a 200% increase in steady state stiffness for a filled structure relative to a hollow structure (Fig. 4A). If the hollow shell allows for fluid flow out of the acinus then the stiffness decreases further.

The increase in stiffness seen in simulation is noticeably larger than in experiment, but there are a number of factors that make a precise correspondence difficult to obtain. The acini exhibit large variations in size, and may be more ellipsoidal than spherical. The choice of shell thickness also plays an important role, since if more material is present, it will lead to a stiffer response. Irrespective of the quantitative differences, the results confirm that the multicellular structure is a major determinant in the mechanical properties of breast acini.

### Multicellular architecture could affect perceived mechanical microenvironment independent of material properties

An advantage of computational modeling is the ability to probe physical variables that might not be accessible by direct measurement. One such variable is the change of perceived stiffness from the perspective of an individual cell within the acinar structures due to lumen filling. If multicellular architecture affects the mechanical response of an acinus, individual cells in the acinus could mechanically sense these differences in structure. Epithelial cells have been shown to mechanosense through cadherin junctions [12], and disrupting these cadherin junctions causes formation of a disorganized, filled structure [13].

**Figure 5:**
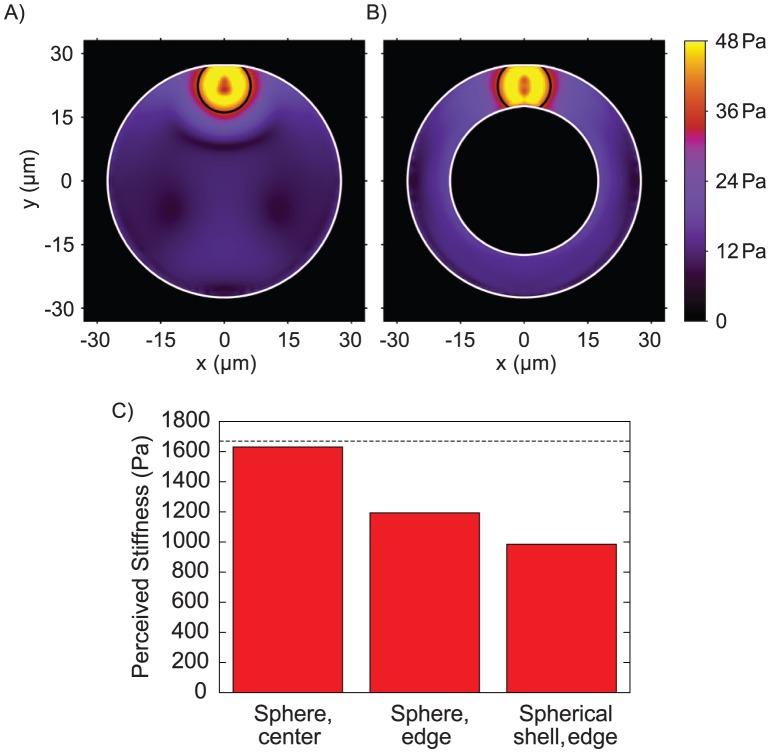
Multicellular architecture could affect the perceived mechanical microenvironment independent of material properties. Cross-section through 3D simulations of single cell contraction in (A) filled and (B) hollow structures, showing the magnitude of shear stress, 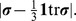 The single cell is shown by the black circle. (C) Perceived stiffness for a single cell in a hollow structure is approximately 20% lower than a filled structure. The dashed horizontal line shows the actual stiffness of the acinus.

We considered a case corresponding to when a cell in an acinus undergoes a very small isotropic contraction. If the cell was embedded within an infinite elastic medium, then the contraction would create a resistive force from the medium, which would be proportional to the medium's stiffness. Thus, the amount of resistive force that the cell experiences in response to a fixed contraction can be used to determine a perceived environmental stiffness (see Simulation Development). To explore this within the simulations, we defined several small volumes within the acini and applied small changes to these volumes as a simple model of cellular contraction. Using our multiphase simulation, we then predicted the force–displacement response of the surrounding structure, from which we can calculate a perceived stiffness. We simulated single cells on the edges of both hollow and filled architectures (Fig. 5A,B) with otherwise identical intrinsic mechanical properties. Our simulation predicts approximately a 20% increase in perceived stiffness due to lumen filling alone (Fig. 5C).

## Discussion

We investigated changes to mechanical properties of a breast epithelial structure during lumen filling. Our data indicate that the filling of the lumen leads to about a 60% increase in stiffness in our AFM measurements. We observed this difference despite MCF10A and MCF10AT cells having very similar mechanical properties, and multicellular structures pre-lumen formation not being detectably different from each other. From these data, we concluded that the arrangement of cells in the acinus affects the mechanical properties of the structure itself. Through numerical simulation, we were able to confirm that the structural differences could indeed explain the increase in the measured AFM stiffness. With the mechanical properties of the simulation calibrated to the experimental data, we then showed that the stiffness that the cells would perceive in the MCF10AT acinus would be increased by 20%. This arises purely due to the altered geometry of tissue structure.

Our results highlight a key role for tissue structure in the mechanosensing at the single cell level. Considering that a two-fold increase in matrix stiffness leads to lumen filling [3], a 20% increase in perceived stiffness due to multicellular structure alone could be a potentially significant step towards loss of structure and function in the mammary gland. In humans, many (but not all) filled-lumen structures progress to form malignant tumors [1]. As increased matrix stiffness drives the malignant phenotype through a contraction-mediated process [3], an increase in perceived stiffness could further destabilize the equilibrium of a multicellular structure. Increases in ECM stiffness of similar magnitude have been shown in mice to promote tumor progression and invasion into the surrounding environment, mediated by integrin force transduction [6].

In order for mechanical changes at cell–cell junctions to be biologically significant, individual cells would have to be capable of mechanosensing through cadherins or other cell–cell junctions. A growing body of evidence suggests that cells can sense mechanical forces through cadherins. The molecular details of how these forces might be transduced is not known [30], but it is clear that transmembrane applied force on E–cadherin results in tension on the actin cytoskeleton through *α*E–catenin [31]. Furthermore, vinculin localizes to E-cadherin when cells are pulled with cadherin-coated beads [12], similar to behavior observed with integrins [32]. Inter-cellular forces of epithelia are relatively large (∼100 nN, similar to cell–ECM forces) and are closely maintained even in the presence of changes in cell morphology and contact area [11]. However, this robust regulation appears to be lost in epithelial-to-mesenchymal transition. Cells increase in area by 20%, adhesion forces drop by much more [33], and signalling pathways may be more susceptible to environmental mechanics, potentially contributing to oncogenesis.

Although the force transduction mechanism is not known, cadherins have been shown to play an important role in morphogenesis and tumor growth. Blocking E-cadherin function in non-malignant breast epithelial cells leads to disorganized, non-polarized structures [13]. This has been previously shown to affect mechanical phenomena such as coherent rotation in breast epithelia [34]. The molecular mechanisms behind cadherin-based mechanosensing are still under investigation, and the techniques described here provide additional tools to study this process.

## Simulation Development

### Development of the simulation framework

The simulations are carried out within a cube, using a right-handed coordinate system in which the *z*-axis points upwards (Figure 4B,C). The cube is filled with a background fluid that is modeled using the Navier–Stokes equations 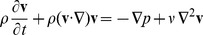
(1)with the incompressibility constraint
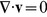
(2)where 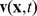 is the fluid velocity, 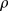 is the density of the fluid, 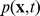 is the fluid pressure, and 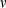 is the fluid viscosity. For the small length scales considered, the term 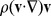 corresponding to the fluid inertia is negligible. This system of equations is simulated using the finite-difference method on a fixed rectangular grid, with the incompressibility constraint imposed via a finite-element projection step [35], [36]. The simulations are written in C++ and carried out on an Apple Mac Pro desktop with dual 2.4 GHz Intel Xeon processors.

The boundary of the acinus is tracked using the level set method [29], whereby an auxiliary function 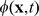 is introduced whose value is the signed distance to the boundary, with 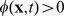 outside the acinus and 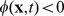 inside the acinus. The function gives an implicit representation of the acinus boundary as the zero contour 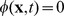, and solves some technical simulation challenges, such as applying boundary conditions at the acinus–fluid interface, and being able to rapidly determine whether a point is inside or outside the acinus by checking the sign of 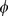.

The acinus is modeled as a linear elastic–viscoelastic solid. Given the small strains of 3% that are considered, we expect that a linear model is a good approximation, and any possible nonlinear behavior can be neglected. Within the acinus, the velocity follows the equation 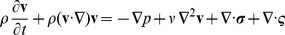
(3)where 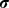 is an elastic stress tensor, and 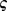 is a viscoelastic stress tensor. Here, we assume that the density and viscosity of the acinus is the same as the fluid. Since we are interested in quasi-static behavior, the viscosity will not play a significant role, and since gravity is negligible at the small scales considered, the relative difference in density will have only a limited effect.

Since the material is incompressible, there is no notion of a bulk modulus due to volumetric deformations, and 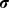 and 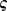 are therefore traceless. For small strains, the two tensors can be updated using the equations 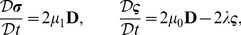
(4)where the derivative 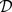 incorporates advection and tensor spin components, and 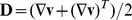 is the rate-of-deformation tensor. Here 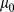 and 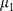 are the viscoelastic and elastic shear moduli respectively, and 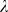 is a viscoelastic damping parameter. Equation 4 has a very similar form to the SLS model, and is a natural three-dimensional extension, with the parameters 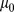, 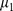, and 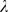 being analogous to 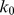, 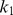, and 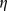 from a SLS one-dimensional linear viscoelastic model.

To inform the simulation with properties based on our measurements, we used a system identification method to fit our creep data to the SLS model. This model (Fig. S2A) consists of a spring (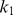) in parallel with a spring–dashpot (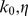). As a check, the parameters obtained from this model (Fig. S2B–S2D) are qualitatively consistent with the data presented in Figs. 2 and 3. While other models may also fit our data, we use the SLS model here simply to inform our simulation with a set of reasonable mechanical parameters.

To carry out the compression of an acinus, a horizontal plate is introduced into the simulation that is free to move in the vertical direction, onto which a constant downward force of 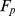 is applied. As it comes into contact with the acinus, it exerts a force on the acinus causing it to deform, until it reaches equilibrium. Figures 4B and 4C show typical snapshots of the simulation for a sphere to model the MCF10AT geometry, and a spherical shell to model the MCF10A geometry. In Figure 4C, four small tubes are placed in the acinus, since the acini in experiments are assumed not to be watertight, and allowing fluid to flow out of the lumen can affect the mechanical response. However, simulations using a watertight central cavity were also carried out.

Using the simulation to quantify the effects of geometry is simplified by the fact that the mechanical model is linear, and that the time scale for the acinus to reach quasi-static equilibrium, 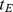, is much smaller than the viscoelastic relaxation time scale 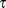. Since the model is linear, if the elastic modulus is scaled by a factor 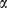, then the force response for a given, fixed displacement will be scaled by 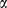 also. Over an intermediate time 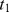, where 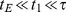, the effective elastic modulus is given by 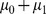, whereas over a much longer time 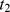 where 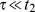, the effective elastic modulus is given by 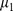. The force response at 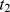 will therefore be equal the force response at 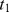 but scaled by a factor of 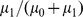. Because of this, it is possible to focus on simulations using elasticity only, setting 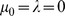. By carrying out several simulations with different displacements, a constant 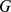 representing a geometrical scaling factor can be obtained, so that 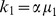. By the above argument, it must also be true that 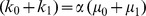 and thus 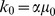.

The simulations are carried out in dimensionless units that are differentiated from their physical counterparts by writing them with a tilde. To connect the simulations to experiments, a mass scale 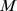, length scale 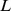, and time scale 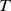 must be introduced, after which any simulation quantity can be related to a physical value by multiplying by the appropriate scales. The simulation cube has side length 3, the acinus has radius 1.1, and the fluid has unit density 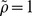. In the MCF10A simulations, the shell has thickness 0.4, which was chosen based on the confocal microscope images in Figure 2. To model a 55 µm diameter acinus, a length scale of 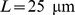 is chosen, and by assuming the density is close to that of pure water, so that 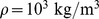, then the mass scale must be 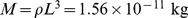.

For each acinus geometry, simulations over a range of plate forces were carried out, using 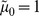 and 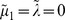. For each simulation, the change in height of the acinus once it has reached equilibrium is recorded. By carrying out a linear fit of the height changes with respect to the plate force, a spring constant 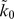 can be calculated. To estimate the shear modulus of the acinus, the value of 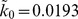 for the solid sphere is compared to the value 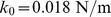 for the MCF10A acinus. Since 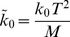
(5)it follows that the time scale is 
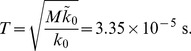
(6)


Hence the shear modulus is 
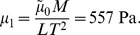
(7)


For an incompressible material where the Poisson ratio is 0.5, the Young's modulus is 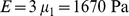. With the physical scales now calibrated, the simulation data of plate force against height change can now be plotted in physical units as in Fig. 3C. This figure gives a value of 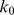 for the MCF10A acinus as 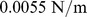. Figure 4D shows plots of pressure in a vertical cross-section through the hollow and filled acini.

### Simulations of perceived stiffness

Suppose first that a single cell is centered at the origin in three-dimensional material that is incompressible with Young's modulus 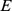, which initially has no stress within it. A spherical region 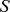 centered on the origin with radius 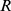 can be introduced, where 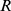 is chosen to be large enough to enclose the cell. Suppose that the cell's volume decreases by a very small amount 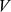. If the radial symmetry is assumed, then it can be analytically derived that the components of the stress tensor 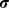 can be expressed in spherical coordinates 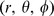 as 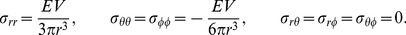
(8)


The total force exerted on the spherical region can therefore be calculated by integrating the radial coordinate of the stress tensor over the surface of sphere 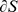 to obtain 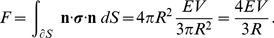
(9)


It therefore follows that force exerted on the cell will be proportional to the shear modulus of the material. This provides a method in which cells can probe their local environment: if a cell contracts by a volume 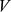 and experiences a total radial force 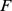, then the perceived shear modulus of the nearby material is given by 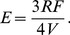
(10)


Using the simulations, we can now address how the effective shear modulus will vary depending on where a cell is situated within a given geometry. To carry this out, we modify the incompressibility condition of Eq. 2 to include a small volume removal, with the form 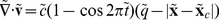
(11)


for 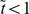 and 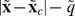. Values of the simulation constants of 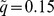, 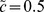, and 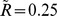 were used, corresponding to a removal of 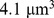 in physical units.

Three simulations carried out for a contraction in the center of a sphere, at the edge of a sphere, and at the edge of a spherical shell. For each one, the effective stiffness that a cell would perceive, using Eq. 10, is shown in Figure 5C. In the center of the sphere, the effective stiffness closely matches the real stiffness of the material, as would be expected for a cell in an infinite medium. However, the stiffness is significantly lessened for the other two simulations, particularly for the spherical shell. While the precise reductions in perceived stiffness are dependent on the parameters used, a marked drop in perceived stiffness and a difference depending on the geometrical configuration of the cells appear to be general features. Using the parameters described here yields a 20% drop in stiffness due to lumen formation alone.

Figures 5A and 5B show plots of the magnitude of the deviatoric stress tensor, computed as 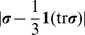, for a contraction at the edge of sphere and spherical shell respectively. This quantity provides a useful scalar measure of shear stress, and for this case is more instructive than examining pressure, given that the analytic solution in Eq. 8 predicts zero pressure. As expected, the shear stresses decay rapidly as a function of distance from the contraction region. Shear stresses are slightly higher for the spherical shell, since it provides less resistance to deformation.

## Methods

### Cell culture

Mammary epithelial cells (MCF10A, Ha-*ras* MCF10AT) were stably transfected with a lentiviral tet-off promoter to express Histone-H2B labeled with eGFP ([37], Addgene plasmid 21210). Following a previously established protocol [20], cells were cultured in DMEM/F12 (UCSF Cell Culture Facility) supplemented with 5% horse serum (Invitrogen), 20 ng/mL EGF (Peprotech), 0.5 µg/mL hydrocortisone (Sigma), 100 ng/mL cholera toxin (Sigma), 10 µg/mL insulin (Sigma) and 1× penicillin–streptomycin (Invitrogen). Cells were passaged using 0.05% trypsin-EDTA (UCSF).

Cells were then fully embedded in laminin-rich, growth-factor reduced extracellular matrix (Matrigel, BD Biosciences) at a concentration of approximately 100 cells/mL using previously described methods [20], [38]. Cells embedded in gels were fed with DMEM/F12 supplemented with 2% horse serum, 5 ng/mL EGF, 0.5 µg/mL hydrocortisone, 100 ng/mL cholera toxin, 10 µg/mL insulin and 1× penicillin–streptomycin. For single cell experiments, cells were extracted from the lrECM gels after twelve hours. For multicellular experiments, structures were extracted either between days 6–8 or days 14–20. Measurements were not noticeably different as a function of number of days in culture.

### Immunofluorescence

Embedded structures fixed as previously described [38]. Structures were pipetted directly onto a glass slide and fixed with 4% paraformaldehyde in phosphate-buffered saline (PBS). Samples were washed with PBS, permeabilized with 1% Triton-X 100, and blocked with 3% BSA in PBS. Samples were stained with anti-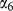-integrin (BD Pharmingen 562473, 1∶500) and mounted with ProLong Gold antifade reagent (Invitrogen). Images were taken on a Yokogawa spinning disk confocal microscope on a Zeiss Axio Observer Z1 using a thermoelectrically cooled Cascade II EMCCD and a 20× 0.4NA objective.

### Extraction from 3D culture

Single cells and multicellular structures were extracted from the lrECM gels for AFM study with an adapted version of previously described acinus-extraction method [38]. The lrECM gels were quickly washed with PBS and then mechanically detached from the culture well. To dissolve the matrix, embedded gels were soaked in a iced PBS-EDTA mixture (0.5 M EDTA pH 8.0 from Invitrogen diluted to 5.5 µM final concentration in PBS) for 10 minutes before being placed in a 1.5 mL tube with excess PBS-EDTA for an additional 25 minutes. The resulting mixture was gently centrifuged at 100–200*g* (single cells 3–5 minutes; acini ∼10 s) and the supernatant was aspirated away. Cells/acini were resuspended in CO_2_-independent media (Invitrogen) with 10% fetal bovine serum and 1× penicillin–streptomycin and plated on a poly-L-lysine-coated (MW>300,000, P5899 Sigma-Aldrich) cover slip for AFM experiments. Poly-L-lysine coatings were used to allow samples to electrostatically attach without activating cell adhesion machinery on the surface.

### Surface preparation

Custom chambers for AFM experiments were made by UV-gluing custom laser-cut acrylic walls (3 mm tall) to a pre-cleaned (KOH base bath) cover slip. Chambers were coated with poly-L-lysine immediately before the experiments by incubating for twenty minutes with a 0.1 mg/mL solution of poly-L-lysine in PBS. Chambers were washed ten times with deionized water and dried with a nitrogen stream before plating samples.

### Atomic force microscopy

AFM experiments were performed on a modified Veeco Bioscope I mounted on a Zeiss Axiovert 25 inverted microscope [39] and a Veeco Catalyst mounted on a Zeiss Axio Observer Z1. Tipless silicon nitride MLCT (30–50 nN/µm, Veeco) cantilevers were used for multicellular experiments, and tipless Arrow cantilevers (10–20 nN/m, Nanoworld) were used for single cell experiments. After a series of initial compression and relaxation steps that ensured good contact between the samples and both the cantilever and glass substrate, two successive compressive force steps of equal size were applied to the sample using a closed-loop piezoelectric. After each force step, the force was maintained for 60 seconds, and deformation and force were recorded as a function of time. Force steps were followed by two similar force-reduction steps, equal in size to the force steps. Data analysis was performed on the first force reduction step. Experiments were performed at 37°C and completed within two hours of plating on poly-L-lysine. There was no discernible change in measured mechanical properties over the course of the experiment. Each sample was also imaged in brightfeld and eGFP epifuorescence (nuclei), and its position on the coverslip was recorded to prevent duplicate testing of the same sample.

### Parameter fitting

Quantification of the compliance of acini and single cells was performed using techniques from system identification. A three-parameter SLS model, as shown in Fig. S2A, is a simple linear viscoelastic system that can capture the observed instantaneous response followed by an exponential decay. We selected an eight-second interval, beginning with the force step, to fit the data to the SLS model using 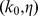 to characterize the viscoelastic response and 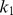 to characterize the elastic response. Values of 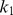 are used to compute the reported stiffnesses.

The parameter fitting was accomplished by first downsampling with a moving average at 5 Hz to filter out high-frequency noise. Next, MATLAB's idgrey was used to solve for the state-space parameters of the first-order ODE for a SLS body, given an initial guess. To ensure a valid solution, the output SLS body was then simulated with the measured force input. The simulated SLS body and actual measured displacements were compared visually to ensure a reasonable fit to the data. Measured responses that could not be fit to an SLS model were discarded, usually due to excessive noise in the measurement.

### Statistical tests

Creep compliances were compared at 8 s time points using *t*-tests as described in the results section with 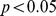 as the significance threshold.

## Supporting Information

Figure S1 **Experimental configuration and typical creep response.** (A) Example image of an MCF10A acinus under a tipless atomic force microscope cantilever. Scale bar 50 µm. (B) Representative creep response of an MCF10A acinus. The thin dotted red line shows a fit to the three-parameter Standard Linear Solid model.
(EPS)

Figure S2 **Standard Linear Solid model parameter values for the experimental data.** (A) Standard Linear Solid model and (B–D) relevant parameters measured by fitting creep curves using system identification techniques. Fit parameters were used to extract mechanical properties for the model. Boxplots are medians extending to the 25th and 75th percentiles. Min/max positions are indicated by the ends of whiskers. Outliers are defined as points beyond 1.5 times the interquartile range from the nearest quartile marker.
(EPS)
